# Effectiveness and safety of endotracheal tube cuffs filled with air *versus* filled with alkalinized lidocaine: a randomized clinical trial

**DOI:** 10.1590/S1516-31802007000600004

**Published:** 2007-11-01

**Authors:** Lais Helena Camacho Navarro, José Reinaldo Cerqueira Braz, Giane Nakamura, Rodrigo Moreira e Lima, Fredson de Paula e Silva, Norma Sueli Pinheiro Módolo

**Affiliations:** Department of Anesthesiology, Faculdade de Medicina de Botucatu, Universidade Estadual Paulista (Unesp), Botucatu, São Paulo, Brazil

**Keywords:** Intratracheal intubation, Cough, Pharyngitis, Hoarseness, Lidocaine, Intubação endotraqueal, Tosse, Faringite, Rouquidão, Lidocaína

## Abstract

**CONTEXT AND OBJECTIVE:**

High intracuff pressure in endotracheal tubes (ETs) may cause tracheal lesions. The aim of this study was to evaluate the effectiveness and safety of endotracheal tube cuffs filled with air or with alkalinized lidocaine.

**DESIGN AND SETTING:**

This was a prospective clinical study at the Department of Anesthesio­logy, Faculdade de Medicina de Botucatu, Universidade Estadual Paulista.

**METHODS:**

Among 50 patients, ET cuff pressures were recorded before, 30, 60, 90 and 120 minutes after starting and upon ending nitrous oxide anesthesia. The patients were randomly allocated to two groups: Air, with ET cuff inflated with air to attain a cuff pressure of 20 cmH_2_O; and Lido, with ET cuff filled with 2% lidocaine plus 8.4% sodium bicarbonate to attain the same pressure. ET discomfort before tracheal extubation, and sore throat, hoarseness and coughing incidence were studied at the time of discharge from the post-anesthesia care unit, and sore throat and hoarseness were studied 24 hours after anesthesia.

**RESULTS:**

Pressures in Lido cuffs were significantly lower than in Air cuffs (p < 0.05). Tracheal complaints were similar for the two groups, except for lower ET discomfort and sore throat incidence after 24 hours and lower systolic arterial pressure at the time of extubation in the Lido group (p < 0.05).

**CONCLUSION:**

ET cuffs filled with alkalinized lidocaine prevented the occurrence of high cuff pressures during N_2_O anesthesia and reduced ET discomfort and postoperative sore throat incidence. Thus, alkalinized lidocaine-filled ET cuffs seem to be safer than conventional air-filled ET cuffs.

## RESUMEN

**CONTEXTO E OBJETIVO:**

Os tubos traqueais são dispositivos utilizados para manutenção da ventilação. A hiperinsuflação do balonete do tubo traqueal, causada pela difusão do óxido nitroso (N_2_O), pode determinar lesões traqueais, que se manifestam clinicamente como odinofagia, rouquidão e tosse. A lidocaína, quando injetada no balonete do tubo traqueal, difunde-se através de sua parede, determinando ação anestésica local na traquéia. O objetivo foi avaliar a efetividade e a segurança do balonete do tubo traqueal preenchido com ar comparado com o balonete preenchido com lidocaína, considerando os desfechos: sintomas cardiovasculatórios (HAS, taquicardia); odinofagia, tosse, rouquidão e tolerância ao tubo traqueal.

**TIPO DE ESTUDO E LOCAL:**

Estudo clínico prospectivo, realizado no Departamento de Anestesiologia da Faculdade de Medicina da Unesp, campus de Botucatu.

**MÉTODOS:**

A pressão do balonete do tubo traqueal foi medida, entre 50 pacientes, antes, 30, 60, 90 e 120 minutos após o início da inalação de N_2_O anestésico. As pacientes foram distribuídas aleatoriamente em dois grupos: Air, em que o balonete foi inflado com ar para obtenção de pressão de 20 cm H_2_O, e Lido, em que o balonete foi preenchido com lidocaína a 2% mais bicarbonato de sódio a 8,4% para obtenção da mesma pressão. O desconforto antes da extubação, e manifestações clínicas como dor de garganta, rouquidão e tosse foram registrados no momento da alta da unidade de cuidados pós-anestésicos, e dor de garganta e rouquidão foram avaliadas também 24 horas após a anestesia.

**RESULTADOS:**

Os valores da pressão no balonete em G2 foram significativamente menores do que os de Air em todos os tempos de estudo, a partir de 30 minutos (p < 0,001). A proporção de pacientes que reagiu ao tubo traqueal no momento da desintubação foi significantemente menor em Lido (p < 0,005). A incidência de odinofagia foi significantemente menor em Lido no primeiro dia de pós-operatório (p < 0,05). A incidência de tosse e rouquidão não diferiu entre os grupos.

**CONCLUSÕES:**

Durante ventilação artificial, empregando-se a mistura de oxigênio e N_2_O, a insuflação do balonete com lidocaína 2% alcalinizada impede que ocorra aumento significante da pressão no balonete e determina maior tolerância ao tubo traqueal e menor incidência de odinofagia no pós-operatório, podendo então ser considerada mais segura e com maior efetividade.

## INTRODUCTION

Endotracheal tubes (ETs) allow pressure to be maintained in the airways during the inhalation phase of artificial breathing and prevent exhalation of regurgitated gastroesophageal contents. However, the pressure of the ET cuff is transmitted to the tracheal mucosa. When elevated, may cause ischemia of the mucosal vessels followed by serious complications such as ciliary loss,^1^ inflammation, ulceration,^2^ hemorrhaging,^3^ tracheal stenosis^4^ and tracheoesophageal fistula.^5^ These are found when the ET cuff pressure is greater than the capillary pressure of the tracheal artery, i.e. 30 cm H_2_O,^6^ which causes tracheal ischemia proportional to the pressure exerted by the cuff and to the length of exposure.^6^

Nitrous oxide (N_2_O), a gaseous anesthetic used in daily anesthetic practice, easily diffuses inside ET cuffs, thereby raising their pressure.^2,7^ Overinflation of the cuff and the consequent tracheal mucosa lesions result in sore throats, hoarseness and coughing, thus causing discomfort to patients after the removal of the intubation.^2,8^

When lidocaine is injected into the ET cuff,^9,10^ it spreads through the semipermeable membrane wall and induces anesthetic action in the trachea. This increases airway tolerance to tracheal^11^ and tracheotomy^12^ tubing. After tracheal extubation, the hemodynamic alterations are minimized, thus reducing the incidence of coughing.^13-17^

Increasing the alkalinity of the local anesthetic using sodium bicarbonate also dramatically increases its diffusion through the ET cuff. This allows the possibility of reducing the dosage of local anesthetic.^18^

## OBJECTIVES

In the literature at our disposal, no reference was found regarding the effects of N_2_O on cuff pressure when the cuff was filled with an alkalinized local anesthetic. The aim of this study was therefore to evaluate the effectiveness and safety of endotracheal tube cuffs filled with air or alkalinized lidocaine during anesthesia using N_2_O. Consequently, the present study compared the pressure in ET cuffs inflated with air or alkalinized lidocaine during anesthesia using N_2_O, and evaluated the presence of cardiocirculatory symptoms such as arterial hypertension and tachycardia, and clinical symptoms such as ET tolerance, coughing, sore throat and hoarseness, following tracheal extubation.

## METHODS

After gaining approval for this study from the Research Ethics Committee of Faculdade de Medicina de Botucatu, Universidade Estadual Paulista, written informed consent was obtained from the patients. We measured ET cuff pressures in 50 female adult patients aged 18 to 65 years who presented American Society of Anesthesiologists (ASA) physical status I and II, with a Mallampatti classification equal to 1. These patients underwent general anesthesia for gynecological surgery (abdominal hysterectomy) or plastic surgery (abdominoplasty, reductive mastoplasty or implantation of silicone prostheses). Patients with tracheotomy, laryngeal disease, laryngeal surgery or a history of smoking, those requiring the insertion of a nasogastric tube and cases in which more than one attempt was needed to achieve tracheal intubation were all excluded.

All the patients received pre-anesthetic medication of midazolam, consisting of a 15 mg dose orally, one hour before anesthesia. After three minutes of pre-oxygenation, anesthesia was induced using propofol (2 mg/kg), sufentanil (0.7 µg/kg) and rocuronium bromide (0.6 mg/kg). Tracheal intubation was always performed by an experienced anesthesiologist, after the neuromuscular blockade had reached its maximum effect, as verified by monitoring the blockade through a sequence of four thumb abductor stimulations (*train-of-four*). No lubricant of any type was used on the tracheal tube before intubation, and all the tracheal tubes utilized had an internal diameter of 7.5 mm.

After orotracheal intubation, the patients were randomly assigned to one of two groups of 25 patients each, according to whether or not lidocaine was used to fill the ET cuff:

### Air group (control):

These patients underwent intubation using a tracheal tube with a large residual volume cuff and low pressure (Rusch, Uruguay), which was initially deflated to its maximum and then inflated with air, up to a pressure of 20 cmH_2_O.

### Lido group (lidocaine):

These patients underwent intubation using a tracheal tube with a large residual volume cuff and low pressure Rusch (Uruguay), which was initially deflated to its maximum and then filled with 2% lidocaine associated with 8.4% sodium bicarbonate, in the proportions of 19.0:1.0 ml, to a volume that was sufficient to cause a cuff pressure of 20 cmH_2_O (15 mmHg).

For the Air group, continuous measurement of the cuff pressure and addition or removal of air from the cuff was achieved using a portable digital manometer pressure-volume gauge (Mallinckrodt, United States) that was connected to the pilot balloon of the tracheal tube. For the Lido group, the cuff pressure was determined by means of a three-way tap connected to the pilot balloon of the tracheal tube and an HP 1290 C pressure transducer (Hewlett-Packard, United States). Introduction or removal of local anesthetic in the cuff was accomplished by connecting a 10 ml glass syringe to one of the outlets of the three-way tap.

Ventilation was controlled by adjusting the current volume and respiratory frequency, so as to maintain the final CO_2_ exhalation pressure (P_ET_CO_2_) between 30 and 35 mmHg. Anesthesia was maintained using isoflurane (minimum alveolar concentration of 0.5 to 1.0), nitrous oxide (0.8 l/min) in oxygen (0.5 l/min), by means of a circuit with CO_2_ absorption and continuous administration of sufentanil (0.005 to 0.01 µg/kg/min). To maintain the neuromuscular blockade, continuous infusion of rocuronium was administered (10 µg/kg/min) by means of a double-channel continuous infusion pump (Abbott, United States), which also served for sufentanil administration.

The patients were monitored using electrocardioscopy (DII derivation), pulse oximetry (SpO_2_), capnometry and non-invasive arterial pressure measurement. They all underwent slow vesicular probing, using a water-based lubricant (KY Gel^®^, Johnson & Johnson) without local anesthetic, in order to facilitate the passage of the probe. The application of local anesthetic by any other means was prohibited, with the exception of what used to inflate the tracheal tube cuff.

During the anesthetic-surgical procedure, four blood samples from peripheral veins were taken from the patients in the Lido group (10, 60 and 120 minutes after tracheal intubation and at the end of anesthesia). These were placed in tubes containing ethylenediaminetetracetic acid (EDTA), in order to analyze the serum concentration of lidocaine using the high-performance liquid chromatography (HPLC) system.

Data relating to the hemodynamic and respiratory characteristics and cuff pressure measurements were obtained after orotracheal intubation but before N_2_O inhalation, and then 30, 60, 90, 120 minutes after the start of N_2_O anesthesia, and again at the end of anesthesia, before stopping the inhalation of N_2_O. At the end of the surgical procedure, the neuromuscular blockade was reverted using neostigmine (0.03 mg/kg) and atropine (0.02 mg/kg), and careful aspiration of the oropharyngeal secretion was performed. Five minutes after reverting the neuromuscular blockade, N_2_O administration was stopped. Controlled ventilation was maintained until signs of deglutition returned and the onset of spontaneous ventilation was achieved, at which point the procedure reverted to assisted ventilation. The tracheal tube was removed when the following signs of complete neuromuscular blockade reversion were seen: response to ulnar stimulation, with fourth stimulus/first stimulus equal to one; spontaneous ventilation, with response to verbal commands (eyes opening or hand squeezing); or movement demonstrating a desire to remove the intubation.

Before removing the N_2_O inhalation, the cuff pressure was determined. The cuff was then totally deflated and the volume of liquid or air was recorded. Next, according to the patient's group allocation, the cuff was reinflated with either air or lidocaine up to the most recently determined pressure, while waiting for the patient to show signs that the parameters for extubation had been fulfilled. At this time the following data were noted: presence of agitation during the period of spontaneous ventilation, duration of anesthesia, time taken for tracheal tube removal and the patient's arterial pressure and heart rate immediately before and after tracheal extubation.

After the end of the anesthetic-surgical procedure, the patients were taken to the post-anesthesia care unit (PACU), where they were evaluated for clinical symptoms relating to the use of the tracheal tube. An independent observer, who did not know which group each patient belonged to, evaluated the presence of excess coughing over a 30-minute period following tracheal extubation and the occurrence of sore throat and hoarseness at the time of release from the PACU and 24 hours after extubation. To evaluate the intensity of the complaints, a visual analog scale was used: 0 (no discomfort) to 10 (the worst possible discomfort).

The patients received postoperative analgesic consisting of dipyrone (1 g) and tramadol (100 mg), injected intravenously at the end of the surgery, and ketoprofen (100 mg, three times/day) intravenously, on the first postoperative day. At the end of the surgical procedure, 4 mg of ondansetron were administered with the intention of establishing antiemesis.

#### Statistical analysis

We calculated that 25 patients would be required in each group, in order to detect a 35% drop in the incidence of coughing and sore throat, for a type I error of 0.05 and a type II error of 0.20, with a power equal to 0.80.

For the variables to have normal distribution in the statistical analysis, parametric tests were used. For anthropometric variables such as the duration of the anesthesia, duration of spontaneous ventilation and arterial pressure and heart rate before and after tracheal intubation, Student's t test was used. The incidence of coughing and the reaction to the tracheal tube were compared using the chi-squared test for multiple variables. All values obtained that did not conform to normal distribution were subjected to nonparametric tests.

The Friedman test was applied to the cuff pressure values in order to compare data within the same group, while the Mann-Whitney test was used to compare values between the two groups. To study the volume of liquid or air injected or removed from the cuff within each group, the Wilcoxon test was used. The differences in the incidence of laryngotracheal symptoms were compared using Fisher's exact test.

For the characteristics that met the presupposed norms, the results were expressed as means and standard deviations of the data obtained. The results for nonparametric characteristics were expressed as the median and first and third quartiles, such that 25% of the observed values were situated in the first quartile, while 75% of the observed values were situated in the third quartile. Values were considered significant when p < 0.05.

## RESULTS

The anthropometric and respiratory characteristics were similar in the two groups (p > 0.05), for example the duration of anesthesia and the time between stopping the use of anesthetic gases and extubation of the patient (p > 0.05). However, the time for which patients remained in spontaneous respiration before fulfilling the parameters for extubation was considerably longer in the Air group (p = 0.047) (Table 1).

**Table 1:** Means and standard deviations of patients’ anthropometric variables and ages, duration of anesthesia, duration of spontaneous ventilation and time until stopping the use of the anesthetic gases prior to extubation, according to group

Variable	Group		p value
	Air	Lido	
Age (years)	45.1 ± 10.2	45.2 ± 9.9	0.80
Weight (kg)	69.2 ± 12.2	65.5 ± 11.2	0.27
Height (cm)	157.8 ± 7.3	157.9 ± 5.7	0.91
Total duration of anesthesia (min)	223.0 ± 72.5	215.2 ± 64.0	0.69
Duration of spontaneous ventilation (min)	12.6 ± 6.1	9.9 ± 2.7	0.047
Time until stopping anesthetic gases (min)	18.6 ± 9.5	15.8 ± 5.6	0.20

The cuff pressures for the Lido group were notably lower than those for the Air group, at all the times studied beyond 30 minutes (p < 0.05). A significant rise in pressure over time occurred only in the Air group (p < 0.05). The cuff pressures in the Air group attained the critical pressure of 30 cmH_2_O after only 30 minutes (Table 2).

**Table 2:** Medians and first and third quartiles relating to cuff pressure (cmH_2_O), according to group and time

	Group		
Time	Air	Lido	Statistical result
M 0	20.00 [20.00; 20.00]	20.00 [20.00; 21.00]	Air = Lido
M 30	30.00 [23.00; 32.00]*†	19.00 [16.75; 26.30]	Air > Lido
M 60	36.00 [30.00; 51.25]*†	21.00 [16.83; 27.60]	Air > Lido
M 90	45.00 [34.00; 63.00]*†	22.40 [14.28; 30.88]	Air > Lido
M 120	48.00 [38.00; 71;25]*†	25.00 [13.48; 27.95]	Air > Lido
M final	48.00 [40.00; 89.00]*†	18.50 [12.15; 32.18]	Air > Lido
Statistical result	M 0 < M 30 < M 60 < M 90 < M 120 = M final	M 0 = M 30 = M 60 = M 90 = M 120 = M final	

M = minute;

* p < 0.05 for the difference between groups at each time;

† p < 0.05 for the difference between times within the same group.

The systolic arterial pressure rose significantly (p < 0.05) in both groups from the moment just before extubation to the moment just after extubation, although the systolic pressure after extubation in the Lido group was notably lower than the pressure in the Air group (p < 0.05). With regard to the diastolic arterial pressure and heart rate at the same times, the two groups performed similarly (p > 0.05).

There was a significant difference regarding the volumes injected into the cuffs, such that larger volumes were injected into the Lido group (p < 0.05). The volumes removed from the cuffs were always larger in the Air group (p < 0.05). In the Air group, there was a notable rise in the volume removed in relation to the volume injected (p < 0.05), while in the Lido group the opposite occurred: the volume removed was always less than what was injected into the cuff (p < 0.05) (Table 3).

**Table 3:** Medians and first and third quartiles relating to the volumes injected (ml) and removed (ml) from tracheal tube cuffs, according to group

	Group		
Time	Air	Lido	Statistical result
Injected	5.0 [4.5; 5.6]*†	6.5 [5.0; 7.8]*	Air < Lido
Removed	8.0 [7.0; 10.0]†	5.0 [3.4; 6.0]*	Air > Lido
Statistical result	Injected < Removed	Injected > Removed	

* p < 0.05 for the difference between groups at each time;

† p < 0.05 for the difference between times within the same group.

**Figure 1:**
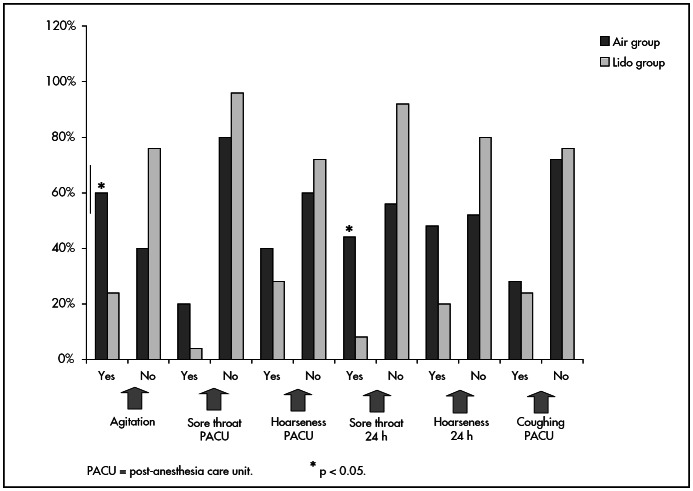
Incidence of agitation at the time of extubation and laryngotracheal morbidity present in the post-anesthesia care unit and 24 hours after extubation, according to group.

The incidence of agitation at the time of tracheal extubation was considerably less in the Lido group (p = 0.022) than in the Air group. In spite of greater incidence of sore throats and hoarseness in the Air group at the time of release from the PACU, there was no notable difference between the groups (p > 0.05). The incidence of sore throats was significantly lower in the Lido group than in the Air group, 24 hours after extubation, while the incidence of hoarseness in the postoperative evaluation did not reveal any notable differences between the two groups (p = 0.07), despite the fact that there was higher occurrence in the Air group. The intensity of the complaints of sore throat and hoarseness revealed little difference between the groups (p > 0.05), while the incidence of coughing during the patients’ stay in the PACU was low and very similar in the two groups (p > 0.05) (Figure 1).

The serum concentration of lidocaine did not differ significantly between the times studied (p > 0.05). It was already detectable 10 minutes after inflating the cuff with local anesthetic and remained constant throughout the study period (Table 4).

**Table 4:** Medians and first and third quartiles relating to the serum lidocaine concentration in the Lido group patients, according to time

Time	Lidocaine concentration (μg/ml)
M 10	1.12 [1.03; 1.31]
M 60	1.08 [1.02; 1.16]
M 120	1.10 [1.04; 1.19]
M final	1.12 [1.02; 1.47]

M = minute; p = 0.84.

## DISCUSSION

The pressure on the pilot balloon of the tracheal tube, which is an indirect measurement of the pressure exerted by the cuff on the tracheal mucosa, is not routinely determined, either by the anesthesiologist or by the doctors and nurses who work in intensive care units.^19-22^ Estimates of the cuff pressure, obtained by squeezing the pilot balloon, may not detect elevated pressure in the cuff.^23^

Several methods have been proposed for minimizing the elevation of cuff pressures during N_2_O anesthesia, such as: use of tracheal tubes with regulatory pressure valves on the cuff, e.g. Brandt's tracheal tube^24,25^ and the Lanz balloon^®^,^26^ which preserve cuff pressure stability but are much too expensive for large scale usage; inflation of the cuff with a mixture of N_2_O/O_2_ in proportions identical to those used in anesthesia,^27^ which presents the possibility of inconvenient deflation, when stopping the inhalation of N_2_O;^27^ use of a tracheal tube with a cuff that is impermeable to N_2_O;^28^ and filling the cuff with 0.9% physiological solution.^2,29^

With regard to inflating the cuff with lidocaine, absence of changes in cuff volume and consequently in cuff pressure is believed to be the main cause of lower postoperative laryngotracheal morbidity. However, in the present study, unlike in studies using physiological solution, continuous pressure readings were made throughout the anesthesia using N_2_O.^11,13,15-18^ The present study makes an important contribution towards clarifying the causes of lower incidence of postoperative laryngotracheal complaints, when using local anesthetic to inflate the cuff.

Initially, lidocaine in chlorohydrate form was used in the cuff, at concentrations of 2%, 4% and 10%, and at doses of 200 to 500 mg.^9-18^ Following that, it was observed that previously warming the lidocaine^13^ and its alkalinization^10,13^ raised the velocity at which it passed through the cuff membrane. This made it possible to reduce the dosage of lidocaine that would achieve the same inflation.^16^ The use of high doses of lidocaine could exceed the serum toxic concentrations of the drug in the event of cuff rupture followed by rapid absorption through the tracheal and bronchial mucosa.^18^

The diffusion mechanism through the cuff membrane can be compared to the mechanism for diffusion in the epidural space.^10^ Increasing the non-ionized fraction of the local anesthetic, through associating it with sodium bicarbonate, results in increased penetration of the drug into the nerve and quicker onset of nerve blockade. It is reasonable to speculate that increasing the non-ionized fraction could precipitate quicker diffusion of the local anesthetic through the cuff.

In experimental studies *in vitro*, it was demonstrated that the basic form of lidocaine was only able to diffuse 65% of the original dosage through the cuff membrane, over a six-hour period. In the chlorohydrate form, only 1% of the lidocaine was able to diffuse.^16^ With alkalinized lidocaine chlorohydrate, the cuff became a reservoir for releasing local anesthetic to the underlying tracheal tissue.^10,16^ In the proportions used in the present study (19.0 ml of 2% lidocaine to 1.0 ml of 8.4% sodium bicarbonate), the pH of the solution changed from 6.92 (lidocaine chlorohydrate) to 7.43 (alkalinized lidocaine), thereby increasing the non-ionized fraction. This enabled rapid passage of lidocaine through the cuff membrane, thus allowing significant lidocaine concentration in the patients’ blood samples just 10 minutes after inflation (1.12 µg/ml). This concentration was comparable to the serum concentrations found at other times of the study, including at the end of anesthesia (1.12 µg/ml) (Table 4). It should be stated that no accumulative effect was detected in the lidocaine serum concentration.

Some precautions must be taken when inflating cuffs containing lidocaine, because of its greater viscosity, in comparison with air or gas. The cuffs must be inflated more slowly than usual, to ensure pressure equilibrium in the system. The elasticity of the cuff tends to force the lidocaine solution to return to the pilot balloon when the inflating pressure is halted and the cuff pressure may consequently fall. One other detail is the need to empty the cuff as fully as possible, as well as removing any air bubbles that accumulate in the cuff at the time of inflation, since these may expand with N_2_O during anesthesia.

Patients undergoing gynecological and plastic surgery were selected for this study because they corresponded to the inclusion or exclusion criteria. Moreover, the procedures included in this study presented similar duration of surgery and could be compared with regard to the total duration of anesthesia without notable differences between the groups (Table 1).

No lubricant was used on the tracheal tube for facilitating its passage past the vocal chords. Cuff lubrication using lidocaine gel or spray has been associated with increased adverse phenomena at the time of awakening from anesthesia induced by tracheal tubes^18^ and may also rupture the cuff.^30^ However, cuff lubrication with water-soluble gel, in association with alkalinized lidocaine, increases tracheal tube tolerance and reduces the incidence of postoperative sore throats.^31^ The use of nasogastric probes during and after surgical operations has also been associated with significantly increased incidence of postoperative sore throats,^32^ which is the reason why their use was among the criteria for excluding patients from the present study.

The behavior of the pressure within the cuff when filled with lidocaine had not previously been studied, and doubts had been raised as to the capacity of lidocaine to reduce laryngotracheal morbidity following extubation. The present study has demonstrated that filling the cuff with lidocaine prevents significant rises in cuff pressure during anesthesia using N_2_O (Table 2).

On the other hand, in the group with the air-inflated cuff, despite an initial pressure that was set well below the critical pressure of 30 cmH_2_O, the cuff pressure equaled the tracheal artery capillary pressure after administering N_2_O for only 30 minutes. Subsequently, it reached high mean values of around 48 cmH_2_O by the end of administering the anesthesia.

Due to the high insolubility of N_2_O in the blood, induced by a low blood-gas partition coefficient, it causes a pressure differential between the blood and the inflated cuff. This is because, while nitrogen diffuses slowly from within the cuff, N_2_O diffuses rapidly to within it, thereby creating the well-documented phenomenon of excessive pressure.^2,7,19,33^

Conflicting results regarding the relationship between high pressure and the occurrence of laryngotracheal morbidity have been obtained.^2,20,34,35^ There seems to be a strong correlation between the incidence of microulceration in the tracheal mucosa area that is in contact with the cuff and the incidence of postoperative sore throats.^2^

Several studies have shown lower incidence of sore throats when the cuff pressure is maintained below 30 cmH_2_O.^2,33,36,37^ Nonetheless, even when maintaining the cuff pressure below 30 cmH_2_O, it is still possible to alter the vein and lymphatic pressures of the trachea, which are, respectively, 16 cmH_2_O and 4 to 6.5 cmH_2_O.^6^ Consequently, cuff pressures greater than these values can cause tracheal mucosa congestion and edema, which will increase the occurrence of clinical symptoms after extubation.

During general anesthesia, lidocaine has been used with the intention of suppressing the cough reflex; as an auxiliary in anesthesia, in order to prevent hemodynamic disturbances during intubation and in the recuperation phase of anesthesia; and in an attempt to prevent postoperative sore throats. To effectively suppress coughing requires a high lidocaine serum concentration^38^ of around 3 µg/ml, which can be achieved through intravenous injection of 1 to 2 mg/kg of the drug.^39^ However, lidocaine administered intravenously can produce sedation and prolong the process of awakening from anesthesia.^18,40^

It has been suggested that sore throats are caused by the activation of tracheal pain receptors.^11^ The proposal of continuous application of local anesthetic to block these nociceptive receptors would therefore seem logical, in an attempt to reduce the incidence of sore throats.

Aside from preventing hyperinflation of the cuff, another advantage of using lidocaine inside the cuff is the reduction in laryngotracheal morbidity. There was a notable increase in tracheal tube tolerance, as demonstrated by the lower incidence of agitation (Figure 1), and also a notably lower rise in systolic arterial pressure at the time of extubation. The significantly lower time needed for spontaneous ventilation in the Lido group (Table 1) is probably due to the greater communication with and collaboration of the patients in this group on awakening from anesthesia. This resulted from less discomfort caused by the cuff while in contact with the trachea, which allowed earlier extubation than in the Air group. There was also a notable reduction in sore throats 24 hours after extubation (Figure 1).

With regard to coughing, there was low incidence with minimal differences between the groups (Figure 1). This was possibly due to the fact that the serum concentration needed for effective prevention of this morbidity was not achieved.

Studies have shown that, with lidocaine used in the cuff, patients present lower incidence of bucking at the time of extubation,^15^ since there is higher tolerance for both tracheal^13-18^ and tracheotomy tubing^12^ and lower incidence of sore throats.^11-18^ Following tracheal extubation, this morbidity appears in 15% to 80% of the cases,^15,18,32^ while in the present study the incidence was 44% in the Air group and 8% in the Lido group (Figure 1). Lidocaine use does not diminish the deglutition reflex, thus confirming the effect that the local anesthetic has on the nociceptive receptors, without the prolonged use of anesthesia, which can cause vocal chord paralysis.^16^

Lidocaine alkalinized with sodium bicarbonate further reduces the incidence of sore throats^13,16,17^ following tracheal extubation, in comparison with cuffs filled with lidocaine.^11,13,15-18^

The frequency of sore throats and hoarseness (Figure 1) at the time of discharge from the PACU was lower in the Lido group, and this continued to be the case regarding the incidence of hoarseness 24 hours after extubation (Figure 1). In spite of the fact that there is some clinical value to these findings of a reduction in patient discomfort, the difference in the results between the two groups did not reach statistical significance.

The results from the present study were irrefutable. This was so much so that, in cases requiring anesthesia using N_2_O in which it is not possible to determine pressure alterations, as in head and neck surgery, the normal routine in our service has been altered such that 2% alkalinized lidocaine for filling the tracheal tube cuff, instead of air, has now become standard practice.

## CONCLUSIONS

During artificial ventilation using a mixture of oxygen and nitrous oxide, inflation of tracheal tube cuffs of large residual volume and low pressure using 2% alkalinized lidocaine, in comparison with cuffs inflated with air, prevented the occurrence of a significant rise in cuff pressure and gave rise to greater tracheal tube tolerance and lower incidence of postoperative sore throats. Aside from that, it promoted a significantly smaller rise in systolic arterial pressure immediately after tracheal extubation.

On the other hand, the incidence of coughing was not reduced and no notable differences in the incidence of sore throats or hoarseness at the time of discharge from the PACU, or in the occurrence of hoarseness during the first 24 hours post-operation, were observed. Also, no significant differences were detected in the diastolic arterial pressure or heart rate at the time of tracheal extubation.

## References

[1] Klainer AS, Turndorf H, Wu HW, Maewal H, Allender P (1975). Surface alterations due to endotracheal intubation. Am J Med.

[2] Combes X, Schauvliege F, Peyrouset O (2001). Intracuff pressure and tracheal morbidity: influence of filling with saline during nitrous oxide anesthesia. Anesthesiology.

[3] Martins RHG, Braz JRC, Bretan O, Figueiredo PR, Defaveri J (1995). Lesões precoces da intubação endotraqueal. [Early injuries of the endotracheal intubation]. Rev Bras Otorrinolaringol.

[4] Berlauk JF (1986). Prolongeal endotracheal intubation vs. tracheostomy. Crit Care Med.

[5] Stauffer JL, Olson DE, Petty TL (1981). Complications and consequences of endotracheal intubation and tracheotomy. A prospective study of 150 critically ill adult patients. Am J Med.

[6] Nordin U (1977). The trachea and cuff-induced tracheal injury. An experimental study on causative factors and prevention. Acta Otolaryngol Suppl.

[7] Stanley TH, Kawamura R, Graves C (1974). Effects of nitrous oxide on volume and pressure of endotracheal tube cuffs. Anesthesiology.

[8] Beebe DS (2001). Complications of tracheal intubation. Semin Anesth Perioperat Med Pain.

[9] Sconzo JM, Moscicki JC, DiFazio CA (1990). In vitro diffusion of lidocaine across endotracheal tube cuffs. Reg Anesth.

[10] Huang CJ, Tsai MC, Chen CT, Cheng CR, Wu KH, Wei TT (1999). In vitro diffusion of lidocaine across endotracheal tube cuffs. Can J Anaesth.

[11] Navarro RM, Baughman VL (1997). Lidocaine in the endotracheal tube cuff reduces postoperative sore throat. J Clin Anesth.

[12] Hirota W, Kobayashi W, Igarashi K (2000). Lidocaine added to a tracheostomy tube cuff reduces tube discomfort. Can J Anaesth.

[13] Huang CJ, Hsu YW, Chen CC (1998). Prevention of coughing induced by endotracheal tube during emergence from general anesthesia--a comparison between three different regimens of lidocaine filled in the endotracheal tube cuff. Acta Anaesthesiol Sin.

[14] Fagan C, Frizelle HP, Laffey J, Hannon V, Carey M (2000). The effects of intracuff lidocaine on endotracheal-tube-induced emergence phenomena after general anesthesia. Anesth Analg.

[15] Altintas F, Bozkurt P, Kaya G, Akkan G (2000). Lidocaine 10% in the endotracheal tube cuff: blood concentrations, haemodynamic and clinical effects. Eur J Anaesthesiol.

[16] Dollo G, Estebe JP, Le Corre P, Chevanne F, Ecoffey C, Le Verge R (2001). Endotracheal tube cuffs filled with lidocaine as a drug delivery system: in vitro and in vivo investigations. Eur J Pharm Sci.

[17] Estebe JP, Dollo G, Le Corre P (2002). Alkalinization of intracuff lidocaine improves endotracheal tube-induced emergence phenomena. Anesth Analg.

[18] Soltani HA, Aghadavoudi O (2002). The effect of different lidocaine application methods on postoperative cough and sore throat. J Clin Anesth.

[19] Braz JR, Navarro LH, Takata IH, Nascimento Júnior P (1999). Endotracheal tube cuff pressure: need for precise measurement. Sao Paulo Med J.

[20] Medalha S, Oliveira LC, Godoy I (1999). Avaliação da pressão no balonete das cânulas endotraqueais e de traqueostomia em pacientes na Unidade de Terapia Intensiva. Rev Bras Terap Intensiva.

[21] Spittle CSN, Beavis SE (2001). Do you measure cuff pressure? A survey of clinical practice. Br J Anaesth.

[22] Vyas D, Inweregbu K, Pittard A (2002). Measurement of tracheal tube cuff pressure in critical care. Anaesthesia.

[23] Fernandez R, Blanch L, Mancebo J, Bonsoms N, Artigas A (1990). Endotracheal tube cuff pressure assessment: pitfalls of finger estimation and need for objective measurement. Crit Care Med.

[24] Brandt L (1991). Prevention of nitrous oxide-induced increases in endotracheal tube cuff pressure. Anesth Analg.

[25] Karasawa F, Okuda T, Mori T, Oshima T (2002). Maintenance of stable cuff pressure in the Brandt tracheal tube during anaesthesia with nitrous oxide. Br J Anaesth.

[26] Navarro LHC, Braz JRC, Pletsch AK, Amorim RB, Módolo NSP (2001). Estudo comparativo das pressões dos balonetes de tubos traqueais contendo ou não válvula reguladora de pressão de Lanz. [Comparative study of tracheal tube pressures with or without Lanz pressure regulation system]. Rev Bras Anestesiol.

[27] Karasawa F, Mori T, Kawatani Y, Ohshima T, Satoh T (2001). Deflationary phenomenon of the nitrous oxide-filled endotracheal tube cuff after cessation of nitrous oxide administration. Anesth Analg.

[28] Fujikawa M, Mizoguchi H, Kawamura J (1995). A new endotracheal tube with a cuff impervious to nitrous oxide: constancy of cuff pressure and volume. Anesth Analg.

[29] Jensen PJ, Hommelgaard P, Sondergaard P, Eriksen S (1982). Sore throat after operation: influence of tracheal intubation, intracuff pressure and type of cuff. Br J Anaesth.

[30] Walmsley AJ, Burville LM, Davis TP (1988). Cuff failure in polyvinyl chloride tracheal tubes sprayed with lignocaine. Anaesthesia.

[31] Estebe JP, Delahaye S, Le Corre P (2004). Alkalinization of intra-cuff lidocaine and use of gel lubrication protect against tracheal tube-induced emergence phenomena. Br J Anaesth.

[32] Bennett MH, Isert PR, Cumming RG (2000). Postoperative sore throat and hoarseness following tracheal intubation using air or saline to inflate the cuff--a randomized controlled trial. Anaesth Intensive Care.

[33] Tu HN, Saidi N, Leiutaud T, Bensaid S, Menival V, Duvaldestin P (1999). Nitrous oxide increases endotracheal cuff pressure and the incidence of tracheal lesions in anesthetized patients. Anesth Analg.

[34] Mehta S, Myat HM (1984). The cross-sectional shape and circumference of the human trachea. Ann R Coll Surg Engl.

[35] Tonnesen AS, Vereen L, Arens JF (1981). Endotracheal tube cuff residual volume and lateral wall pressure in a model trachea. Anesthesiology.

[36] Mandoe H, Nikolajsen L, Lintrup U, Jepsen D, Molgaard J (1992). Sore throat after endotracheal intubation. Anesth Analg.

[37] Suzuki N, Kooguchi K, Mizobe T, Hirose M, Takano Y, Tanaka Y (1999). [Postoperative hoarseness and sore throat after tracheal intubation: effect of a low intracuff pressure of endotracheal tube and the usefulness of cuff pressure indicator]. Masui.

[38] Yukioka H, Yoshimoto N, Nishimura K, Fujimori M (1985). Intravenous lidocaine as a suppressant of coughing during tracheal intubation. Anesth Analg.

[39] Gonzalez RM, Bjerke RJ, Drobycki T (1994). Prevention of endotracheal tube-induced coughing during emergence from general anesthesia. Anesth Analg.

[40] DiFazio CA, Neiderlehner JR, Burney RG (1976). The anesthetic potency of lidocaine in the rat. Anesth Analg.

